# N-Myristoyltransferase 2 Is Regulated by Polyphosphate-Driven Lysine Interactions

**DOI:** 10.3390/biom16081096

**Published:** 2026-07-27

**Authors:** Isabella Martins, Kaia Bailey, Anni Ge, Ethan Belrose, Xiaolong Yang, Zongchao Jia

**Affiliations:** 1 Department of Biomedical and Molecular Sciences, Queen’s University, Kingston, ON K7L 3N6, Canada; 2 Department of Pathology and Molecular Medicine, Queen’s University, Kingston, ON K7L 3N6, Canada

**Keywords:** polyphosphate, NMT2, myristoylation, lysine repeats, lysine polyphosphate modification, KPM, phosphorylated Src

## Abstract

Inorganic polyphosphate (polyP) is a ubiquitous polymer increasingly recognized as a regulator of protein function through lysine polyphosphate modification (KPM), a reversible interaction with lysine-rich protein regions. Although numerous KPM targets have been identified, the functional consequences of polyP binding remain poorly understood. N-myristoyltransferase 2 (NMT2), an essential enzyme that catalyzes protein N-myristoylation and regulates membrane-associated signaling pathways, was previously identified as a candidate KPM target. Here, we investigated the molecular and functional relationship between polyP and NMT2. Using a fluorescence-based coenzyme A release assay, we found that polyP directly down-regulated NMT2 enzymatic activity in a dose-dependent manner, with long-chain polyP (polyP_700_) producing significantly greater inhibition than medium-chain polyP (polyP_100_). In cells, both exogenous polyP treatment and induction of endogenous polyP synthesis reduced phosphorylation of Src, a downstream signaling protein whose activation depends on N-myristoylation, supporting inhibition of NMT2 function in vivo. PolyP also decreased the viability of NMT2-overexpressing HeLa cells in a dose-dependent manner, suggesting functional consequences for cellular fitness. Biochemical analysis further revealed that polyP induced a pronounced electrophoretic mobility shift in NMT2 that was completely reversed by a 25-residue lysine-rich competitor peptide, consistent with a specific and reversible interaction mediated through lysine-enriched regions of the enzyme. These findings identify NMT2 as a functional target of polyP and demonstrate that polyP negatively regulates NMT2 activity, downstream Src signaling, and cellular viability. Our results expand the emerging concept of lysine polyphosphate modification and establish polyP as a previously unrecognized regulator of protein lipidation-dependent signaling pathways.

## 1. Introduction

Inorganic polyphosphate (polyP) is a highly conserved biopolymer composed of linear chains of phosphate residues linked by high-energy phosphoanhydride bonds [1]. Present across all domains of life, polyP participates in a wide range of biological processes, including phosphate storage, stress adaptation, energy metabolism, blood coagulation, and bacterial virulence [2,3]. Beyond these traditional roles, growing evidence demonstrates that polyP can directly regulate protein function through specific protein–polyP interactions.

Recent studies have identified a novel form of protein regulation termed lysine polyphosphate modification (KPM), in which polyP interacts with lysine-rich regions of proteins in a stable, non-covalent manner [4]. KPM has been shown to alter protein electrophoretic mobility and influence biological activity in both bacterial and mammalian systems [4]. Importantly, several proteins involved in signaling and virulence have been identified as KPM targets, suggesting that polyP may function as a broader regulatory molecule than previously appreciated [4,5]. One mammalian candidate identified through KPM screening is human N-myristoyltransferase 2 (NMT2) [4], an enzyme responsible for catalyzing N-myristoylation, a lipid modification critical for protein localization, membrane association, and intracellular signaling [6,7]. Dysregulation of NMT activity has been implicated in numerous diseases, including cancer and infectious disorders, highlighting the importance of understanding mechanisms that regulate NMT2 function [7,8].

Notably, human NMT2 contains lysine-rich regions (sequence: 46-KKKKKKQKRKKEK-58) that exhibit strong polyP responsiveness, suggesting that polyP may directly influence its activity. Despite increasing interest in polyP-mediated protein regulation, the functional consequences and molecular basis of KPM remain poorly understood, particularly in mammalian systems. This study therefore investigates the interaction between polyP and NMT2 to better define how polyP influences protein function and signaling. We hypothesized that inorganic polyP directly interacts with lysine-rich regions of NMT2 through a reversible KPM-like mechanism, thereby modulating NMT2 activity and downstream signaling. Based on the strong polyP-dependent electrophoretic mobility shift observed during KPM screening [4], we predicted that polyP association would inhibit NMT2 enzymatic function in a chain length-dependent manner, reduce Src phosphorylation in cells by impairing N-myristoylation-dependent signaling, and alter cellular fitness in NMT2-overexpressing cells. We further hypothesized that disruption of this interaction by a lysine-rich competitor peptide would reverse the polyP-induced mobility shift, supporting a specific lysine-mediated mechanism of regulation. Understanding this relationship will contribute to the broader characterization of polyP as a multifunctional regulator in eukaryotic biology.

## 2. Materials and Methods

### 2.1. Protein Expression and Purification

Recombinant proteins were expressed in *E. coli* BL21 (DE3) transformants. Starter cultures were grown for 18–20 h at 37 °C in Terrific Broth (1:100 dilution; BioShop, Burlington, ON, Canada, Cat#TER409) supplemented with appropriate antibiotics, shaking at 180 rpm. These cultures were then used to inoculate fresh media and grown until an OD600 of approximately 0.6 was reached. Protein expression was induced with 1 mM IPTG (BioShop, Burlington, ON, Canada, Cat#IPT002), after which cultures were incubated for an additional 18 h at 20 °C.

Cells were harvested by centrifugation at 4000 rpm for 30 min and resuspended in lysis buffer (150 mM NaCl, 50 mM Tris-HCl, 3 mM β-mercaptoethanol, and 5% glycerol) containing lysozyme (100 µg/mL). Cell lysis was further achieved by sonication using a Branson Sonifier for 7 min at 35% amplitude with a pulse cycle of 5 s on and 15 s off. Cellular debris was removed by centrifugation at 18,000 rpm for 30 min, and the clarified supernatant was applied to amylose resin (New England Biolabs, Ipswich, MA, USA, Cat#E8021L) pre-equilibrated with lysis buffer. The lysate was incubated with the resin for 30 min at 4 °C with gentle agitation.

The resin was loaded onto a gravity-flow column, and non-specifically bound proteins were removed by washing with lysis buffer (three column volumes). MBP-tagged proteins were eluted using 10 mM maltose. Protein purity was assessed by SDS-PAGE (9% acrylamide/bis-acrylamide) followed by Coomassie Brilliant Blue G-250 staining.

A secondary purification step was performed using fast protein liquid chromatography (FPLC) with a Superdex 200 Increase 10/300 GL size-exclusion column (Cytiva, Marlborough, MA, USA, Cat#28990944) equilibrated in lysis buffer. Proteins were eluted at a flow rate of 1 mL/min, and 2 mL fractions were collected for further analysis.

### 2.2. N-Myristoylation Fluorescence Assay

Enzymatic activity assays were performed in 96-well microplates (Invitrogen, Carlsbad, CA, USA, Cat#M33089) in a total reaction volume of 115 µL per well. Each reaction contained 10 µL DMSO (10% *v*/*v* in water), 25 µL of 30 µM myristoyl-CoA (Millipore Sigma, Oakville, ON, Canada, Cat#M4414), and purified NMT2 enzyme at a final concentration of 6.3 nM (50 µL per well). The fluorescent probe 7-diethylamino-3-(4-maleimido-phenyl)-4-methylcoumarin (CPM; Abcam, Waltham, MA, USA, Cat#AB145275) was included at a final concentration of 8 µM (10 µL per well). Reactions were supplemented with 5 µL of either assay buffer (20 mM potassium phosphate, pH 8.0, 0.5 mM EDTA, 0.1% Triton X-100, and 0.7% DMSO [10% in water]), polyP, or the NMT2 inhibitor Zelenirstat (MedChemExpress, Monmouth Junction, NJ, USA, Cat#HY-147308), depending on the experimental condition. A 100 mM stock solution of long-chain polyP (~700 phosphate units; polyP_700_) and medium-chain polyP (~100 phosphate units; polyP_100_) was prepared by dissolving 10.2 mg of polyP_700_ or polyP_100_ in 1 mL of sterile water (Kerafast, Newark, CA, USA, Cat#EUI003 and Cat#EUI005). Reactions were initiated by the addition of 15 µL of 30 µM Hs pp60src peptide substrate (Biomatik, Kitchener, ON, Canada). Fluorescence was monitored over 30 min with readings taken at 1 min intervals, using an excitation wavelength of 380 nm and emission detection at 470 nm [9].

### 2.3. Western Blotting

Protein samples were separated via electrophoresis using SDS-PAGE gels run at 300 V for 30 min. Following electrophoretic separation, proteins were transferred onto PVDF membranes using a wet transfer system at 25 V for 8 h at 4 °C. Transfer efficiency and total protein loading were assessed by staining membranes with Ponceau S solution (ThermoFisher Scientific, Waltham, MA, USA, Cat#A40000278). Following visualization, membranes were rinsed with 1× Tris-buffered saline (TBS; 20 mM Tris-HCl, 150 mM NaCl, pH 7.4) and blocked overnight at 4 °C under continuous agitation in TBS containing 0.05% Tween-20 (TBST) supplemented with 5% (*w*/*v*) non-fat dry milk. Membranes were subsequently washed 3 times with TBS for 5 min per wash and incubated with the appropriate primary antibody diluted in blocking buffer for 18 h at 4 °C with continuous agitation. After primary antibody incubation, membranes were washed three times with TBST for 5 min each and then incubated with the corresponding secondary antibody for 1 h at room temperature under continuous agitation. For phosphorylated Src detection, rabbit phospho-Src family (Tyr416) primary antibody (Cell Signalling Technology, Danvers, MA, USA, Cat#2101; RRID:AB_331697) in a 1/1000 dilution and goat anti-rabbit DyLight800 (Invitrogen, Carlsbad, CA, USA, Cat#SA5-35571; RRID:AB_2556775) in a 1/10,000 dilution were used.

Immunoblot band intensities were quantified using Fiji (ImageJ2). Phosphorylated Src signal intensities were normalized to the corresponding β-actin loading control for each lane. Normalized values from three independent biological replicates were expressed relative to the untreated control and are presented as mean ± standard deviation (SD).

### 2.4. Cell Culture and Transfection for HeLa and PPK-Inducible Cell Lines

HeLa cells and engineered PPK-inducible mammalian cell lines were maintained in Dulbecco’s Modified Eagle Medium (DMEM) supplemented with 10% fetal bovine serum. We generated a PPK-inducible HeLa cell line, and induction of polyP synthesis in this system was achieved by treating cells with doxycycline at concentrations ranging from 0.75 to 1.25 µg/mL. NMT2-eGFP plasmid transfections were carried out using Lipofectamine™ 3000 following the manufacturer’s instructions (Thermo Fisher Scientific, Waltham, MA, USA).

### 2.5. Establishment of a Doxycycline-Inducible PPK1 HeLa Cell Line

Subcloning of PPK1 into pTRIPZ: Full-length PPK1 cDNA from *Pseudomonas aeruginosa* UCBPP-PA14 (GenBank genome accession No. CP000438.1) was PCR-amplified and cloned into the AgeI and MluI sites of the pTRIPZ lentiviral vector (Open Biosystems/Horizon Discovery) to generate FLAG–PPK1–pTRIPZ. An N-terminal FLAG tag was introduced via the forward primer. To prevent cleavage by AgeI, a silent nucleotide substitution was incorporated into the reverse primer to disrupt an internal AgeI site near the 3′ end of the coding sequence. Primers contained AgeI and MluI restriction sites, and the FLAG-tag coding sequence was included in the forward primer. The PCR product was digested with AgeI and MluI, ligated into pTRIPZ, and transformed into *E. coli* Stbl3 cells, and positive clones were confirmed by restriction digestion.

Forward (AgeI–FLAG–PPK1-F):

5′-ATACCGGTACCATGGACTACAAAGACGATGACGACAAGAATACGCAGCAAGGAC TT GATG-3′

Reverse (MluI–PPK1-R):

5′-GTAACGCGTTCAACGTGCGGTAAGCACCGGCGCGGCCAGCTTCTCCAGCAGGGT CGCCTGGGTATTGCGGGGGTTCTGGTTTCCGGTGGGACTGAGCCG-3′

Lentivirus production: HEK293T cells were seeded on poly-L-lysine-coated 35 mm dishes and grown to ~80% confluency. Cells were transfected with 0.5 μg FLAG-PPK1-pTRIPZ, 0.38 μg psPAX2, and 0.13 μg pMD2.G using 3 μL PolyJet reagent. After 24 h, medium was replaced with DMEM/10% FBS containing 10 mM sodium butyrate to enhance viral production. Viral supernatants were collected at 48 and 72 h, pooled, filtered through a 0.45 μm low-protein-binding filter, aliquoted, snap-frozen, and stored at −80 °C.

Lentiviral infection and selection: HeLa cells were seeded at 6 × 10^4^ cells/well in a 12-well plate. Cells were infected with increasing volumes of lentiviral supernatant (0–499.2 μL/well) in the presence of 8 μg/mL polybrene, while maintaining a constant final volume. After 24 h, cells were selected with 1 μg/mL puromycin, with medium refreshed every 2–3 days. The culture receiving the highest virus dose that yielded healthy surviving colonies was expanded, while the uninfected well served as a kill control. Doxycycline-inducible FLAG-PPK1 expression was confirmed by Western blotting after treatment with 1 μg/mL doxycycline for 24–48 h.

### 2.6. Metabolic Activity Assay

Cells were seeded in triplicate in a flat-bottom 96-well plate (Sarstedt, Saint-Leonard, QC, Canada) and cultured until reaching approximately 80% confluency. Cells were then transfected as described above. Following transfection, polyP_700_ was added to the appropriate wells at final concentrations of 1, 2, or 5 mM. Metabolic activity was assessed by adding 10 µL of metabolic activity reagent (Abcam, Waltham, MA, USA, Cat#AB228554) per 100 µL of culture medium, followed by incubation for 4 h at 37 °C. Absorbance was subsequently measured at 460 nm, with background correction performed using readings at 620 nm. Equal numbers of viable cells were counted and seeded into each well prior to treatment to ensure comparable initial cell densities across all experimental conditions.

### 2.7. NuPAGE Competition Assay

Recombinant NMT2 protein was purified to 2.8 mg/mL and incubated in vitro with long-chain polyP (polyP_700_) at indicated concentrations: 1, 2, or 5 mM. Samples were incubated for 30 min at room temperature to allow for the protein–polyP interaction. For competition experiments, a 25-amino-acid poly-lysine (poly-K) peptide (Biomatik, Kitchener, ON, Canada) was added either alone or following pre-incubation of NMT2 with polyP_700_ at varying molar ratios. Reactions were incubated for an additional 30 min. All samples were subsequently mixed with 2× Laemmli buffer in a 1:1 ratio and heated for 5 min at 95 °C.

Samples were resolved using pre-cast 4–12% Bis-Tris NuPAGE gels (ThermoFisher Scientific, Waltham, MA, USA, Cat#NP0336BOX), electrophoresed at 200 V for 45 min, and visualized by Coomassie staining. Electrophoretic mobility shifts were assessed by comparing the apparent molecular weight of NMT2 across treatment conditions. A polyP-induced upward shift was interpreted as altered protein migration consistent with polyP association, while restoration of canonical migration following poly-K treatment was interpreted as competitive displacement of polyP from lysine-rich regions of NMT2. All experiments were performed in at least three independent replicates to ensure reproducibility.

## 3. Results

### 3.1. Polyphosphate Inhibits NMT2 Myristoylation Activity

**Figure 1:**
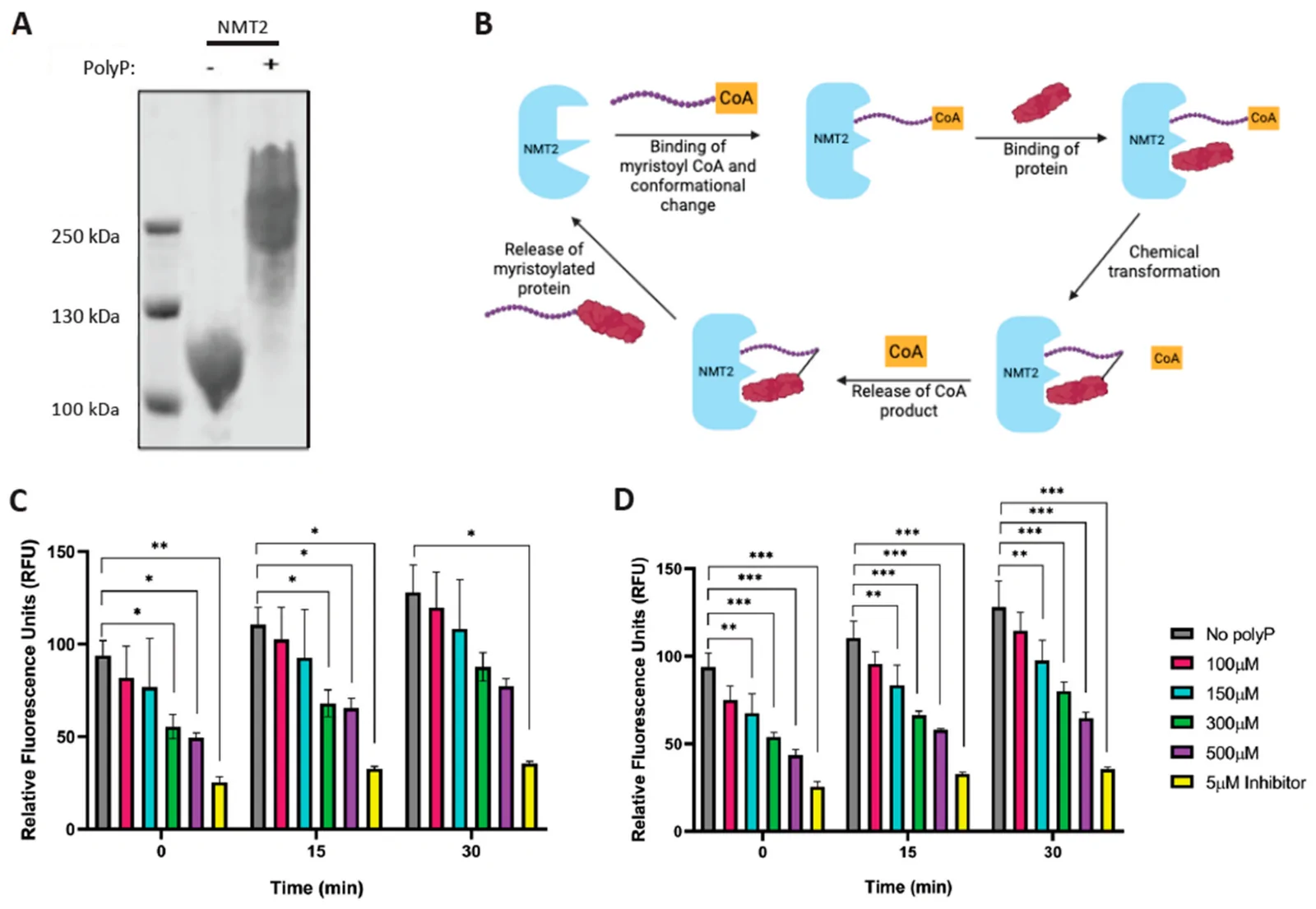
Polyphosphate induces an electrophoretic mobility shift and inhibits NMT2 myristoylation activity in vitro. (**A**) NuPAGE analysis of purified MBP-tagged NMT2 expressed from a custom HT25 bacterial expression vector. Addition of 5 mM polyP_700_ induces a pronounced upward electrophoretic mobility shift [4]. (**B**) Schematic overview of the fluorescence-based NMT2 activity assay. During N-myristoylation, NMT2 transfers a myristoyl group from myristoyl-CoA to the N-terminal glycine residue of a substrate protein, releasing free CoA. The fluorescent probe CPM [7-diethylamino-3-(4-maleimido-phenyl)-4-methylcoumarin] reacts with liberated CoA to generate a fluorescent signal proportional to enzymatic activity. (**C**) PolyP_100_ inhibits NMT2 activity in a concentration-dependent manner from 100 to 500 μM. (**D**) PolyP_700_ similarly suppresses NMT2 activity, with stronger inhibition observed at increasing concentrations from 100 to 500 μM. A 5 μM NMT2 inhibitor was included as a positive control for enzymatic inhibition. Statistical significance was determined by two-way ANOVA using three biological replicates. Error bars represent ± standard deviation. *, *p* < 0.05; **, *p* < 0.01; ***, *p* < 0.001.

N-myristoyltransferase 2 (NMT2) catalyzes the transfer of a 14-carbon myristoyl group to N-terminal glycine residues of substrate proteins, a modification essential for membrane localization and downstream signaling [6,10]. Following its identification as a lysine polyphosphate modification (KPM) target based on a pronounced polyP-dependent electrophoretic mobility shift (Figure 1A), NMT2 was selected for functional characterization [4].

To determine whether polyP influences NMT2 enzymatic activity, an in vitro fluorescence-based assay was performed using the probe 7-diethylamino-3-(4-maleimido-phenyl)-4-methylcoumarin (CPM), which detects free coenzyme A (CoA) released during myristoyl transfer (Figure 1B) [9]. Fluorescence intensity therefore served as a direct readout of NMT2 activity. Two polyP chain lengths were examined, polyP_100_ and polyP_700_, to assess potential chain length-dependent effects.

Addition of polyP_100_ resulted in a concentration-dependent reduction in CoA release, indicating inhibition of NMT2 activity (Figure 1C). The inhibitory effect was most pronounced at 500 μM polyP_100_ and remained reduced relative to untreated controls across all time points examined. PolyP_700_ produced a stronger inhibitory response, with 150–500 μM concentrations significantly suppressing NMT2 activity throughout the assay period (Figure 1D). Compared with polyP_100_, the longer polyP_700_ polymer consistently exhibited greater inhibition, suggesting that polyP-mediated suppression of NMT2 is influenced by polymer length.

To benchmark these effects, reactions containing a standard NMT2 inhibitor were included as a reference control. Collectively, these findings demonstrate that polyP directly inhibits NMT2 activity in vitro, with long-chain polyP exerting the strongest effect.

### 3.2. Polyphosphate Reduces Phosphorylated Src Levels in Cells

**Figure 2:**
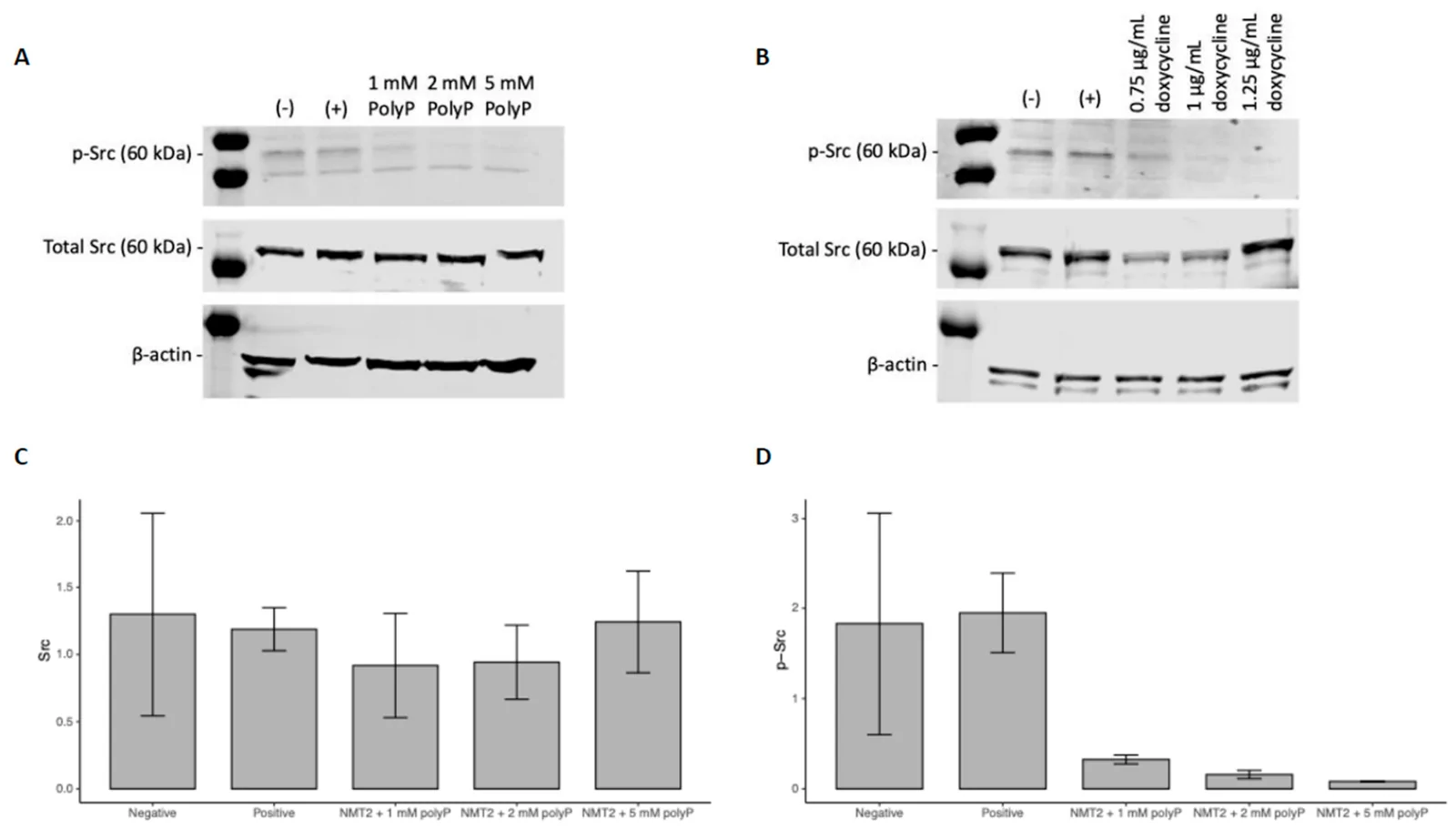
Polyphosphate selectively reduces Src phosphorylation without altering total Src protein levels. (**A**) Representative Western blot analysis of phosphorylated Src (p-Src), total Src, and β-actin in HeLa cells under the indicated conditions: untransfected cells (−), cells overexpressing NMT2 (+), and NMT2-overexpressing cells treated with 1, 2, or 5 mM polyP_700_. Increasing concentrations of exogenous polyP_700_ resulted in a dose-dependent reduction in p-Src levels. (**B**) Representative Western blot analysis of p-Src, total Src, and β-actin in PPK-inducible HeLa cells. Cells were either untransfected (−), overexpressing NMT2 (+), or overexpressing NMT2 with induction of endogenous polyP synthesis using 0.75, 1.0, or 1.25 μg/mL doxycycline. Increasing doxycycline concentrations similarly resulted in reduced p-Src levels. β-actin was used as a loading control. Blots shown are representative of three independent experiments. (**C**) Densitometric analysis of total Src normalized to β-actin from A, showing no significant change across treatments. (**D**) Densitometric analysis of p-Src normalized to β-actin from A, demonstrating a concentration-dependent decrease following polyP_700_ treatment. β-actin served as a loading control. Data are representative of three independent experiments ((**C**,**D**) mean ± SD, n = 3).

To determine whether polyP-mediated inhibition of NMT2 extends to a cellular context, downstream Src signaling was examined in WT HeLa and polyphosphate kinase (PPK)-inducible HeLa cells (Figure 2). Src was selected as a functional readout because N-myristoylation is required for its membrane localization and kinase activation [11]. Cells were transfected with eGFP-tagged NMT2 and treated either with exogenous polyP_700_ or doxycycline to induce endogenous polyP synthesis in PPK-expressing cells.

In WT HeLa cells, increasing concentrations of exogenous polyP_700_ produced a dose-dependent reduction in phosphorylated Src (p-Src) levels, with the greatest decrease observed at 5 mM polyP_700_ (Figure 2A). In contrast, total Src protein levels did not show a corresponding decrease across all treatment conditions, indicating that polyP specifically reduced Src phosphorylation rather than overall Src expression. Densitometric analysis of p-Src and total Src normalized to β-actin confirmed these findings, demonstrating a progressive decrease in p-Src with increasing polyP_700_ concentrations while total Src levels remained relatively constant (Figure 2C,D).

Similarly, induction of endogenous polyP production in PPK cells resulted in progressively lower p-Src levels as doxycycline concentrations increased (Figure 2B). Total Src protein levels remained unchanged across all induction conditions, indicating that intracellularly generated polyP likewise selectively suppresses Src phosphorylation rather than Src expression.

We demonstrate here that both exogenous and intracellularly generated polyP suppress Src phosphorylation in cells while maintaining relatively stable total Src protein levels. Given the dependence of Src activation on N-myristoylation, these findings support a model in which polyP negatively regulates NMT2 activity, resulting in reduced Src phosphorylation without altering total Src abundance.

**Figure 3:**
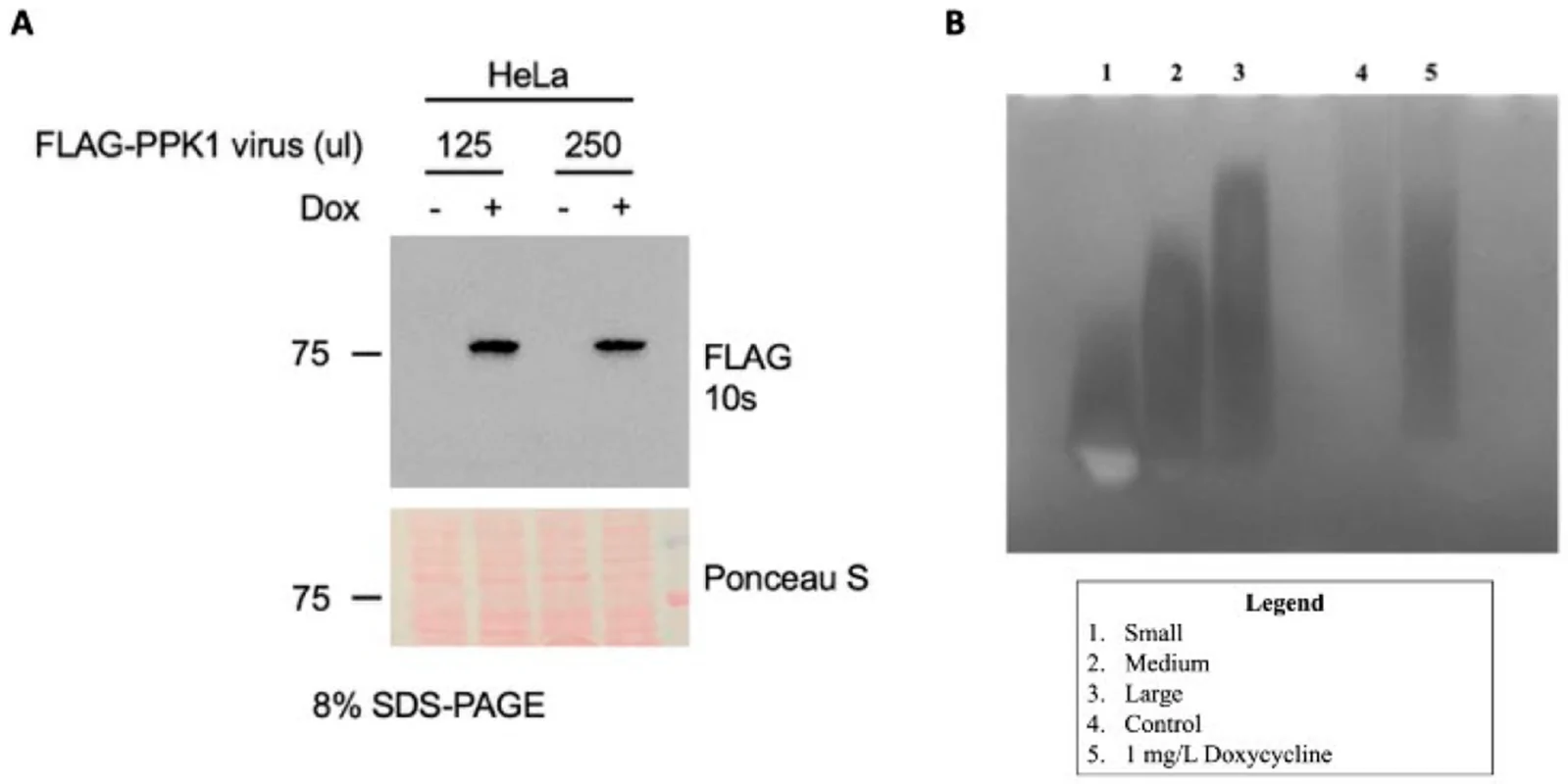
Validation of doxycycline-inducible FLAG-PPK1 expression and polyP production in HeLa FLAG-PPK1/pTRIPZ cells. (**A**) Cells were maintained under selection with 0.25 µg/mL puromycin and treated with or without doxycycline (1 µg/mL) for 24 h to induce FLAG-PPK1 expression. Whole-cell lysates were collected and analyzed via Western blotting to confirm doxycycline-dependent expression of FLAG-PPK1. (**B**) PolyP production was further assessed using a 1.5 mm urea–polyacrylamide gel, with polyP isolated from induced cells compared against small-, medium-, and long-chain polyP standards.

To validate the inducible expression system, HeLa FLAG-PPK1/pTRIPZ cells were treated with doxycycline (1 μg/mL) for 24 h, and FLAG-PPK1 expression was assessed by Western blotting. FLAG-PPK1 protein was readily detected in doxycycline-treated cells but was absent in untreated controls, confirming tight doxycycline-dependent regulation of transgene expression (Figure 3A). To determine whether FLAG-PPK1 expression resulted in polyphosphate synthesis, polyP was isolated from induced cells and analyzed by urea–polyacrylamide gel electrophoresis. Distinct polyP species were detected in induced cells and migrated within the range of polyP standards of varying chain lengths, confirming successful intracellular polyP production following FLAG-PPK1 induction (Figure 3B).

### 3.3. Polyphosphate Reduces Viability of NMT2-Overexpressing HeLa Cells

**Figure 4:**
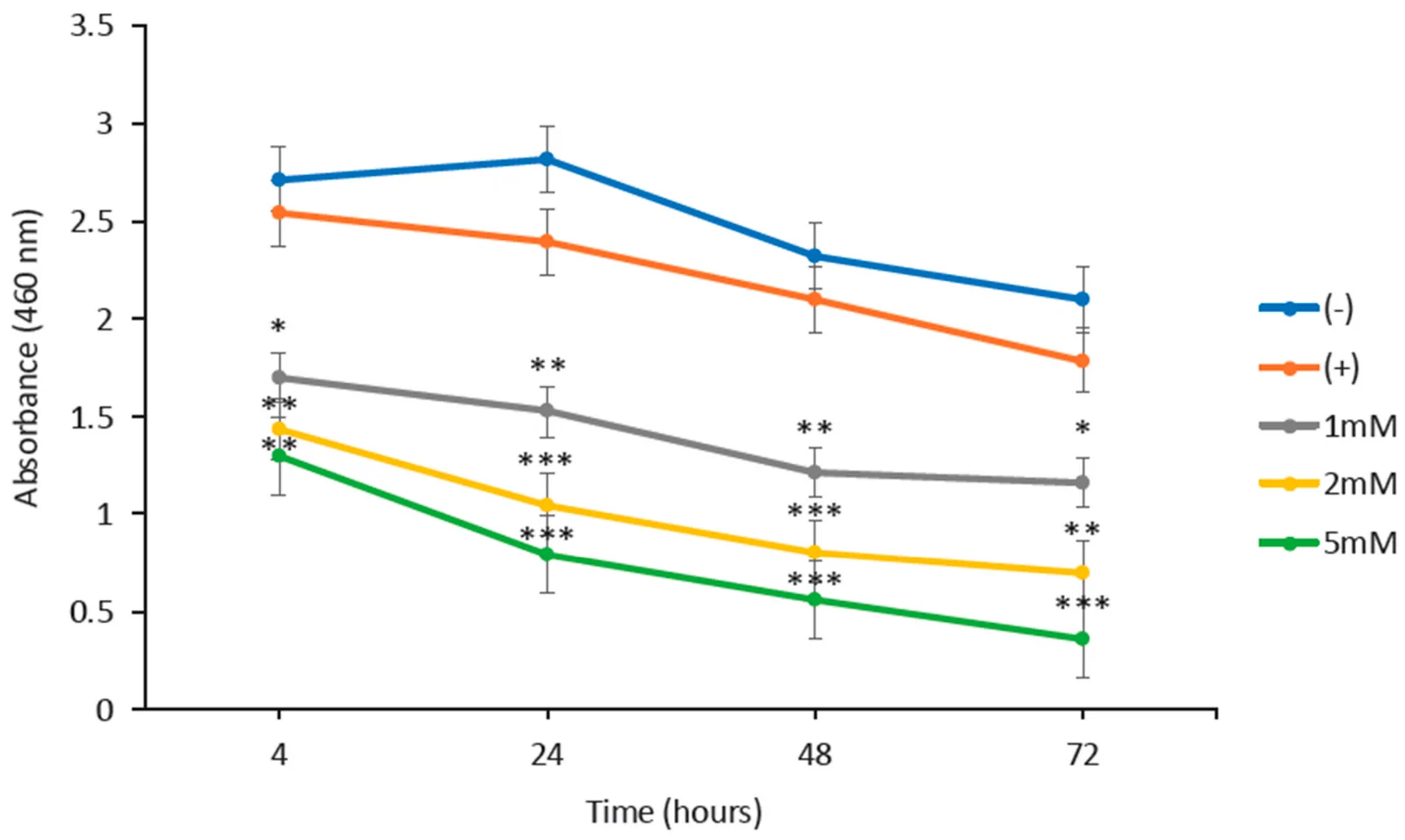
PolyP_700_ reduces viability of NMT2-overexpressing HeLa cells in a dose-dependent manner over 72 h. HeLa cells transiently expressing eGFP-tagged NMT2 were treated with 1, 2, or 5 mM of polyP_700_ and monitored for viability over a 72 h period using a metabolic assay. Untransfected HeLa cells without treatment served as the negative control (blue line), while NMT2-transfected cells without polyP treatment served as the transfection control (orange line). Grey, yellow, and green lines correspond to NMT2-transfected cells treated with 1, 2, and 5 mM of polyP_700_, respectively. PolyP treatment produced a concentration-dependent reduction in viability, with 2 and 5 mM of polyP_700_ causing the greatest decreases relative to untreated controls. Absorbance was measured at 460 nm and normalized to background readings collected at 620 nm. Data represent mean ± standard deviation from three independent experiments. Statistical analysis was performed using two-factor ANOVA with replication followed by Tukey’s multiple-comparison test; significance is shown only for comparisons between each treatment group and the untransfected control (−). *, *p* < 0.05; **, *p* < 0.01; ***, *p* < 0.001.

To assess whether polyP affects cellular fitness in the context of NMT2 overexpression, HeLa cells transiently expressing eGFP-tagged NMT2 were treated with increasing concentrations (1–5 mM) of long-chain polyP (average chain length ~700 Pi units) and monitored for up to 72 h using a metabolic viability assay (Figure 4). Untransfected HeLa cells (blue curve, Figure 4) and NMT2-transfected cells without polyP (orange curve) served as controls.

NMT2 overexpression alone caused a modest, non-significant decrease in viability compared to untransfected cells, consistent with minimal baseline toxicity. In contrast, the addition of polyP produced a clear, dose-dependent reduction in activity across all concentrations tested (Figure 4). The effect was most pronounced at 5 mM polyP, where a substantial decrease in signal was already evident at 24 h and persisted through 72 h.

Notably, the metabolic activity curves for polyP-treated cells ran largely parallel to the controls after the initial 24 h time point, indicating that polyP primarily reduced the metabolic signal early in the treatment period rather than causing a progressive decline throughout the assay. Because the metabolic assay measures overall cellular metabolic activity, these data do not distinguish between reduced proliferation and increased cell death. Together, these data demonstrate that polyP treatment reduces the metabolic activity of NMT2-overexpressing cells, supporting a functional association between polyP exposure, NMT2 modulation, and altered cell fitness.

To determine whether these effects reflected a general response of HeLa cells to polyP or were enhanced by NMT2 overexpression, untransfected HeLa cells were treated with identical concentrations of polyP700 (1, 2, and 5 mM) and analyzed using the same metabolic assay (Figure S5). Under these conditions, polyP treatment alone produced comparatively modest changes in metabolic activity over the 72-h period. Cells treated with 1 and 2 mM of polyP700 exhibited only slight reductions in metabolic signal relative to untreated controls, whereas 5 mM of polyP700 slowed the increase in metabolic signal without causing a progressive decline over time. These findings suggest that the more pronounced reduction in metabolic activity observed in NMT2-overexpressing cells is not solely attributable to the general effects of the polyP treatment.

### 3.4. A Poly-Lysine Peptide Abolishes the polyP-Induced NMT2 Mobility Shift

**Figure 5:**
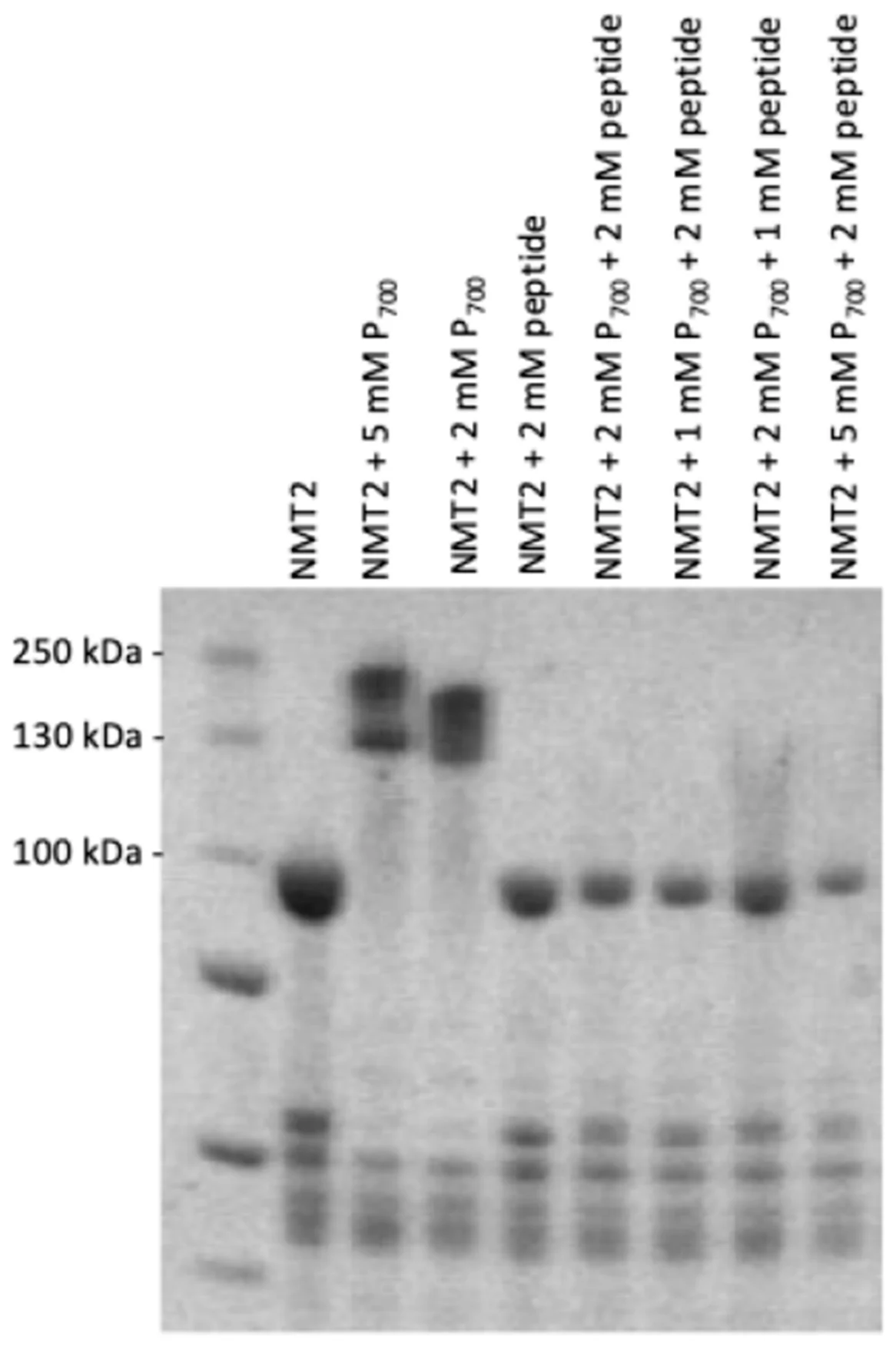
A poly-lysine peptide abolishes the polyP-induced electrophoretic mobility shift in NMT2. NuPAGE analysis of purified MBP-tagged NMT2 following incubation with polyP_700_ and/or a 25-residue poly-lysine (poly-K) peptide. Increasing concentrations of polyP induced a pronounced upward electrophoretic mobility shift in NMT2. Subsequent addition of the poly-K peptide abolished the polyP-induced shift across multiple polyP-to-peptide ratios, restoring migration to the expected molecular weight. These findings support competitive disruption or displacement of polyP binding to lysine-rich regions within NMT2. Gels were stained with Coomassie Brilliant Blue.

To determine whether a lysine-rich peptide could disrupt the interaction between polyP and NMT2, purified MBP-tagged NMT2 was analyzed by NuPAGE following incubation with polyP and/or a 25-residue poly-lysine (poly-K) peptide (Figure 5). Untreated NMT2 migrated at its expected molecular weight of approximately 100 kDa, consistent with the MBP fusion protein. Incubation with polyP_700_ induced a pronounced upward electrophoretic mobility shift in a concentration-dependent manner, with 5 mM polyP producing the strongest effect.

Treatment with the poly-K peptide alone did not alter NMT2 migration, indicating that the peptide itself does not affect protein mobility. However, the addition of the peptide following polyP incubation completely abolished the polyP-induced shift. Restoration of normal migration occurred not only under equimolar conditions but also across multiple non-equimolar polyP-to-peptide ratios.

These findings show that the polyP-induced NMT2 mobility shift is reversible and can be effectively disrupted by a lysine-rich competitor peptide, supporting the specificity of the interaction between polyP and lysine-enriched regions within NMT2.

## 4. Discussion

This study identifies N-Myristoyltransferase 2 (NMT2) as a functional target of polyP and demonstrates that polyP modulates both its biochemical activity and downstream cellular signaling. Our data support a model in which polyP acts as a regulatory polymer capable of directly influencing NMT2 function through lysine-dependent interactions consistent with lysine polyphosphate modification (KPM). A key finding of this work is that polyP induces a pronounced electrophoretic mobility shift in NMT2, consistent with a strong interaction between the polymer and the protein. This shift was concentration-dependent and reversible upon addition of a lysine-rich competitor peptide, suggesting that polyP binding is mediated primarily through electrostatic interactions with lysine-enriched regions rather than irreversible covalent modification. The ability of a poly-lysine peptide to fully abolish the shift across multiple stoichiometric ratios further supports a competitive binding mechanism and reinforces the importance of lysine density in mediating polyP association with NMT2. These observations align with emerging models of KPM as a reversible, non-covalent regulatory mechanism rather than a classical post-translational modification.

Functionally, polyP was found to inhibit NMT2 enzymatic activity in vitro using a fluorescence-based CoA release assay. Both medium-chain (polyP_100_) and long-chain (polyP_700_) polymers reduced NMT2 activity in a dose-dependent manner; however, inhibition was significantly stronger and more sustained with polyP_700_. This chain length dependence is consistent with the known physicochemical behavior of polyP, where longer polymers exhibit increased charge density, multivalent binding capacity, and structural flexibility, enabling more stable interactions with protein surfaces [12]. The observed suppression of CoA production indicates that polyP reduces NMT2 enzymatic activity rather than affecting the assay chemistry itself, supporting a direct functional consequence of polyP binding. Since CoA is released as a direct product of the myristoylation reaction, reduced CoA generation reflects decreased enzymatic activity [10]. However, the present study does not distinguish between potential mechanisms of inhibition. PolyP may influence NMT2 function through one or more mechanisms, including altered substrate accessibility, allosteric effects, or other changes in enzyme dynamics. Additional biochemical and structural studies will be required to define the molecular basis of inhibition. Given the demonstrated interaction between polyP and NMT2, these findings support a model in which polyP binding disrupts catalytic function, potentially through electrostatic interactions that alter active-site accessibility or induce conformational changes that reduce enzymatic efficiency.

Importantly, these in vitro findings translated into a cellular context. Both exogenous polyP treatment and endogenous polyP induction via a PPK-inducible system resulted in reduced levels of p-Src, a well-established downstream reporter of N-myristoylation-dependent membrane localization and activation [13,14]. In contrast, total Src protein levels remained unchanged under all treatment conditions, indicating that polyP selectively affects Src activation rather than Src expression. Quantitative analysis of the HeLa cell experiments further confirmed a concentration-dependent decrease in p-Src while demonstrating stable total Src levels following polyP treatment. Given that Src requires N-myristoylation for proper membrane targeting and full kinase activation, the observed reduction in p-Src strongly supports the conclusion that polyP impairs NMT2 function in cells [11,13]. The convergence of both pharmacological (exogenous polyP) and genetic (PPK induction) approaches strengthens the physiological relevance of this effect and indicates that intracellular polyP levels are sufficient to modulate NMT2-dependent signaling pathways [15].

In addition to suppressing downstream Src signaling, polyP also reduced the metabolic activity of NMT2-overexpressing HeLa cells in a dose-dependent manner. While NMT2 overexpression alone produced only a modest and non-significant reduction in metabolic activity [16,17], polyP treatment caused a marked early reduction in metabolic signal, particularly at higher concentrations of polyP_700_. The largely parallel trajectories of the curves after the initial 24 h period suggest that polyP primarily altered the initial metabolic response rather than producing a continuously increasing effect over time. Since the assay measures metabolic activity rather than cell number or viability directly, these data cannot distinguish between reduced proliferation and increased cell death. Nevertheless, these findings further support the functional significance of polyP-mediated NMT2 inhibition in cells and suggest that disruption of N-myristoylation-dependent signaling may contribute to impaired cellular growth or survival [18].

We determined whether these effects reflected non-specific responses to polyP by additionally examining untransfected HeLa cells exposed to identical concentrations of polyP. Under our experimental conditions, polyP alone produced comparatively modest changes in metabolic activity, with 1 and 2 mM polyP causing only slight reductions in metabolic signal and 5 mM polyP slowing the increase in metabolic activity over time rather than producing a progressive loss of signal. These findings suggest that the more pronounced response observed in NMT2-overexpressing cells cannot be explained solely by polyP-induced effects that are independent of NMT2, although future studies directly comparing these conditions using complementary assays will be important to establish the contribution of NMT2 more definitively.

The in vitro and cellular data provide complementary evidence that polyP negatively regulates NMT2 activity and downstream signaling outputs. Mechanistically, our biochemical data support a direct interaction between polyP and lysine-rich regions within NMT2, as demonstrated by the electrophoretic mobility shift and its reversal by a lysine-rich competitor peptide. While the interaction is associated with reduced enzymatic activity, the present experiments do not establish how polyP binding inhibits catalysis. Whether inhibition results from altered substrate binding, allosteric regulation, conformational changes, or another mechanism remains to be determined. The reversibility of the interaction further suggests that polyP functions as a dynamic regulator rather than an irreversible inhibitor, consistent with its broader role as a modulatory polyanion in cellular stress and metabolic responses [1,19].

Our findings extend the emerging concept of lysine polyphosphate modification to a central enzyme in lipidation-dependent signaling pathways [20]. While previous studies have largely focused on nuclear or bacterial proteins, the identification of NMT2 as a polyP-responsive enzyme broadens the scope of KPM into membrane-associated signaling processes [21,22]. Given the essential role of NMT2 in protein targeting and cellular signaling networks [7,8], polyP-mediated inhibition may have widespread downstream consequences beyond Src, potentially affecting multiple myristoylation-dependent substrates.

Our biochemical data reveal that NMT2 inhibits its enzymatic activity in a chain length-dependent manner and suppresses downstream signaling in cells. These results position polyP as a previously unrecognized regulator of protein lipidation and expand the functional landscape of polyP beyond its classical roles in phosphate storage and stress adaptation [23]. Although our biochemical experiments using purified recombinant NMT2, including electrophoretic mobility shift, lysine-competition, and enzymatic activity assays, support a direct interaction between polyP and NMT2, future structural and biophysical studies will be valuable for defining the molecular interface and quantitatively characterizing the interaction. Techniques such as isothermal titration calorimetry (ITC), surface plasmon resonance (SPR), or microscale thermophoresis (MST) could provide complementary information regarding binding affinity, kinetics, and the contribution of polyP chain length to complex formation [24], although the heterogeneous polyP polymer would make the quantitative analysis very challenging, if not impossible.

Several limitations should be considered. The exact structural basis of the polyP–NMT2 interaction remains unresolved, and whether polyP directly occludes the active site or induces allosteric changes is not yet clear [25,26]. Additionally, while Src phosphorylation provides a useful functional readout of NMT2 activity, it remains an indirect measure and may be influenced by additional regulatory pathways [27]. Nevertheless, the unchanged total Src protein levels observed under all treatment conditions indicate that the decrease in p-Src reflects altered Src activation rather than reduced Src expression.

An additional limitation relates to the concentrations of polyP used in this study. Although the concentrations employed are consistent with those commonly used to investigate polyP-mediated protein regulation in vitro, the physiological concentration and subcellular distribution of polyP in mammalian cells remain incompletely defined [28]. PolyP exists as heterogeneous polymers that are compartmentalized within cells, making accurate determination of local concentrations challenging [29]. Consequently, the effective concentration encountered by proteins such as NMT2 may differ substantially from bulk cellular measurements. Future studies aimed at quantifying intracellular polyP pools and defining the concentration thresholds required for NMT2 regulation under physiological conditions will be important for establishing the biological relevance of this mechanism.

Another limitation of the present study is that the metabolic assay provides an indirect measure of cellular fitness and does not distinguish between cytostatic and cytotoxic effects. Future studies incorporating complementary assays, including direct cell counting, proliferation assays, and markers of apoptosis or necrosis, will be valuable for defining the cellular mechanisms underlying the observed reduction in metabolic activity. In addition, although equal numbers of viable cells were seeded across all experimental groups prior to treatment, transient NMT2 overexpression produced a modest, non-significant reduction in baseline metabolic activity. While this difference was not statistically significant, future studies using stable or inducible expression systems may help determine whether elevated NMT2 expression alone influences cellular metabolism.

Future work will be required to define the structural interface between polyP and NMT2, determine whether other myristoylated proteins are similarly affected, and establish the physiological conditions under which intracellular polyP reaches concentrations sufficient to regulate NMT2 in vivo [30,31].

## 5. Conclusions

This study demonstrates that inorganic polyP directly regulates N-myristoyltransferase 2 (NMT2), identifying NMT2 as a functional target of polyP-mediated protein regulation. We show that polyP associates with lysine-rich regions of NMT2 through a reversible interaction consistent with lysine polyphosphate modification (KPM), induces a characteristic electrophoretic mobility shift, and inhibits NMT2 enzymatic activity in a chain length-dependent manner. Long-chain polyP exhibited the strongest inhibitory effects, suggesting that polymer length is an important determinant of regulatory potency.

Importantly, the biochemical effects of polyP translated into a cellular context, where both exogenous and endogenous polyP reduced Src phosphorylation without altering total Src protein levels, supporting the selective inhibition of Src activation rather than changes in Src expression. These findings support a model in which polyP negatively regulates NMT2-dependent signaling pathways and influences cellular fitness through modulation of protein myristoylation.

Our results expand the emerging concept of lysine polyphosphate modification beyond previously characterized targets and establish polyP as a previously unrecognized regulator of protein lipidation. Given the central role of NMT2 in membrane targeting and signal transduction, polyP-mediated regulation may have broad implications for cellular signaling networks, stress responses, and disease-associated pathways. Future studies aimed at defining the structural basis of the polyP–NMT2 interaction and identifying additional polyP-sensitive myristoylated proteins will further clarify the biological significance of this regulatory mechanism.

## Data Availability

The data that support the findings of this study are available from the corresponding author upon reasonable request.

## Supplementary Materials

The following supporting information can be downloaded at: https://www.mdpi.com/article/10.3390/biom16081096/s1, Figure S1: Full, uncropped western blot images corresponding to the representative blots presented in Figure 2A; Figure S2: Full, uncropped western blot images corresponding to the representative blots presented in Figure 2B; Figure S3: Full raw images corresponding to Figure 3; Figure S4: Uncropped Coomassie-stained NuPAGE gel corresponding to Figure 5; Figure S5: Effect of polyP700 on metabolic activity of untransfected HeLa cells over 72 h.
