# Supplementary material for: N-Myristoyltransferase 2 Is Regulated by Polyphosphate-Driven Lysine Interactions
Source: Biomolecules. 2026 Jul 27;16(8):1096. doi: 10.3390/biom16081096 (PMC13510580; doi:10.3390/biom16081096)
Supplement: Supplementary file 1 [file biomolecules-16-01096-s001.zip › biomolecules-4395242-supplementary.pdf]

SUPPLEMNTAL MATERIAL

N-Myristoyltransferase 2 is Regulated by Polyphosphate-Driven Lysine Interactions

Isabella Martins[1], Kaia Bailey[1], Annie Ge[2], Ethan Belrose[1], Xiaolong Yang[2] and Zongchao Jia[1]

[1]Department of Biomedical and Molecular Sciences, Queen's University, Kingston, Ontario, Canada K7L 3N6

[2]Department of Pathology and Molecular Medicine, Queen's University, Kingston, Ontario, Canada K7L 3N6

Correspondence should be addressed to: jia@queensu.ca

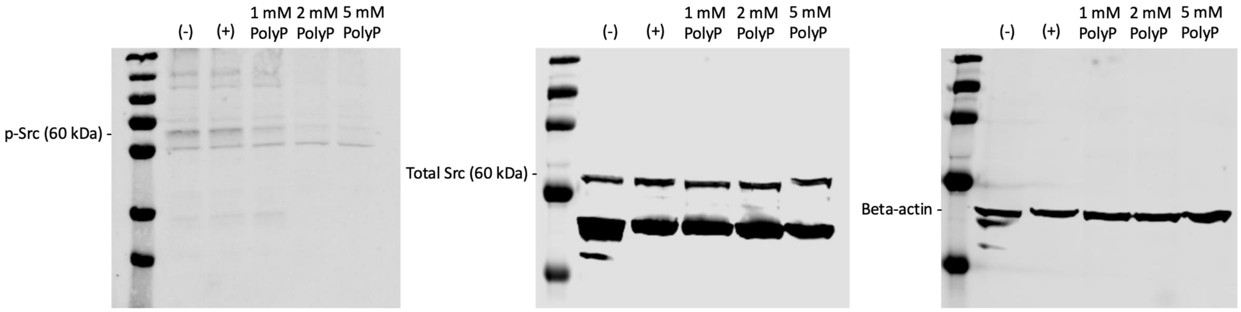

Figure S1. Full, uncropped western blot images corresponding to the representative blots presented in Figure 2A. HeLa cells were either untransfected (-), transiently overexpressing eGFP-tagged NMT2 (+), or overexpressing NMT2 and treated with increasing concentrations of polyP700 (1, 2, or 5 mM). Membranes were probed for phosphorylated Src (p-Src), total Src, and β-actin. The regions shown in figure 2A are indicated.

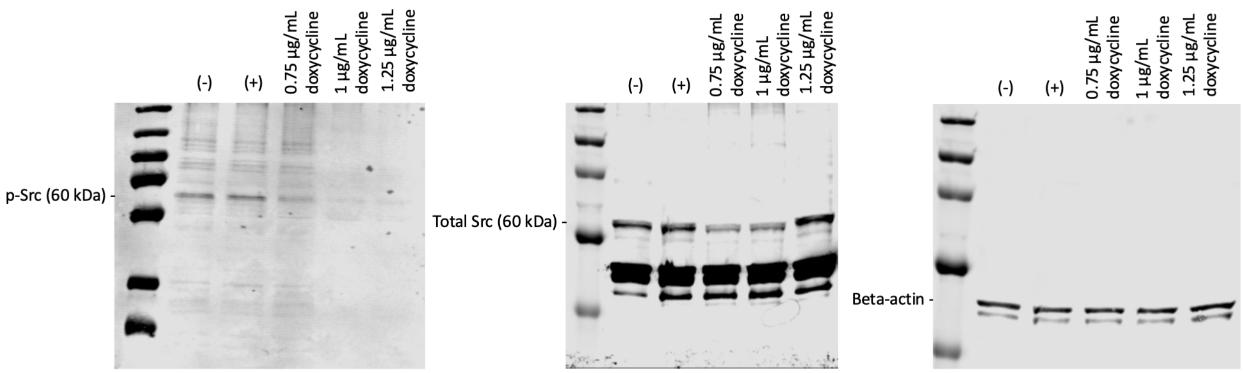

Figure S2. Full, uncropped western blot images corresponding to the representative blots presented in Figure 2B. PPK-inducible HeLa cells were either untransfected (-), transiently overexpressing eGFP-tagged NMT2 (+), or overexpressing NMT2 with induction of endogenous polyP synthesis by treatment with doxycycline (0.75, 1.0, or 1.25 µg/mL). Membranes were probed for phosphorylated Src (p-Src), total Src, and β-actin. The regions shown in Figure 2B are indicated.

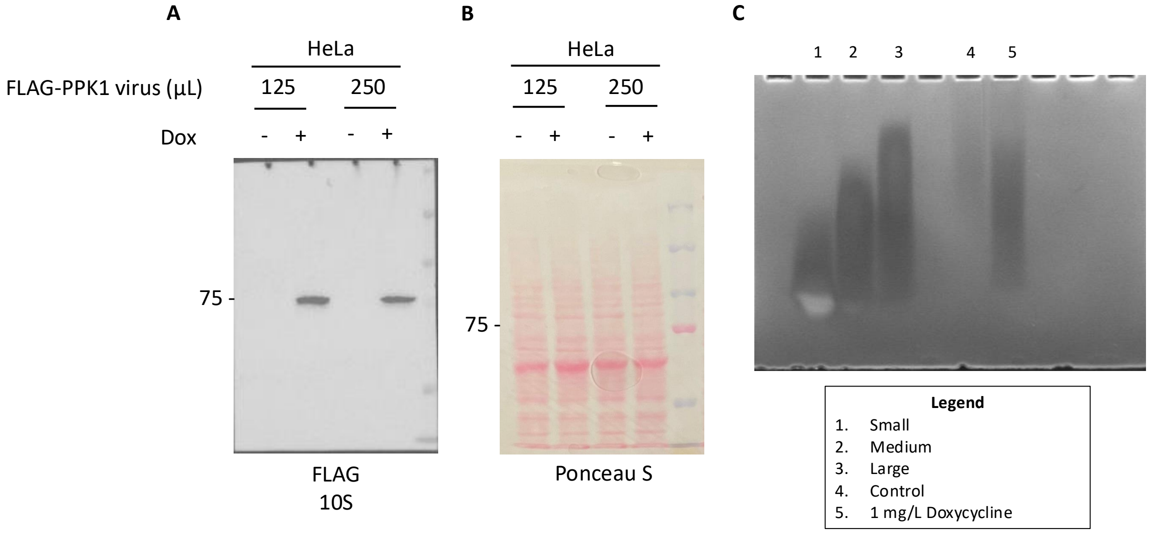

Figure S3. Full raw images corresponding to Figure 3. A) Uncropped western blot validating doxycycline-inducible FLAG-PPK1 expression in HeLa FLAG-PPK1/pTRIPZ cells maintained under puromycin selection (0.25 µg/mL) and treated with or without doxycycline (1 µg/mL) for 24 hours, corresponding to Figure 3A. B) Uncropped Ponceau S-stained membrane showing total protein loading for the western blot presented in Figure 3A. C) Uncropped 1.5 mm urea-polyacrylamide gel showing polyP isolated from doxycycline-induced cells alongside small-, medium-, and long-chain polyP standards, corresponding to Figure 3B.

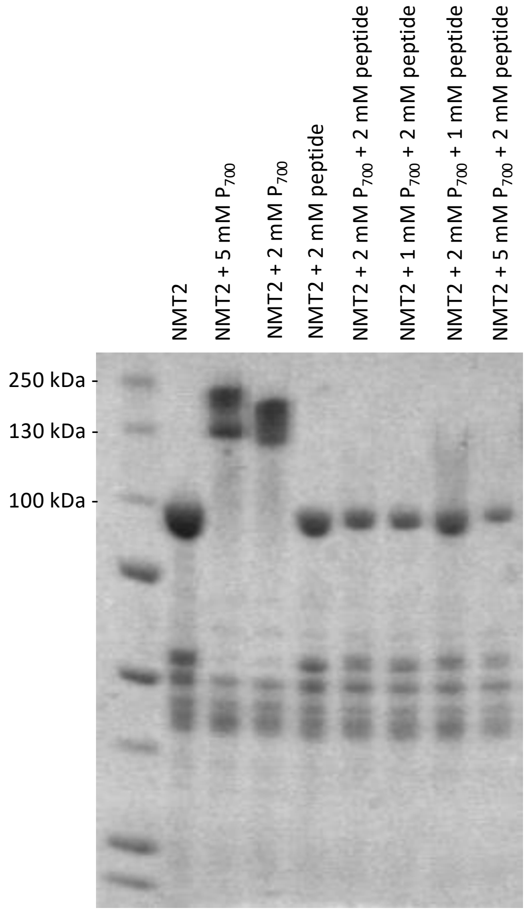

Figure S4. Uncropped Coomassie-stained NuPAGE gel corresponding to Figure 5. Purified MBP-tagged NMT2 was incubated with increasing concentrations of polyP700 and/or a 25-residue poly-lysine (poly-K) peptide prior to electrophoresis. PolyP700 induced a pronounced upward electrophoretic mobility shift of NMT2, whereas subsequent addition of the poly-K peptide abolished the polyP-induced shift across multiple polyP-to-peptide ratios, restoring migration to the expected molecular weight. The uncropped gel is shown to document the complete experimental dataset corresponding to the cropped image presented in Figure 5.

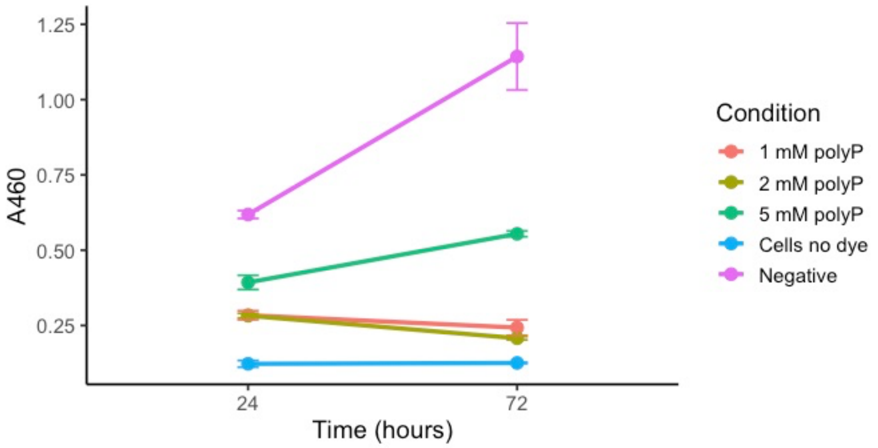

Figure S5. Effect of polyP$_{700}$ on metabolic activity of untransfected HeLa cells over 72 hours. Untransfected HeLa cells were treated with 1 mM (orange), 2 mM (dark yellow), or 5 mM (green) of polyP$_{700}$ and monitored using the same metabolic viability assay employed in Figure 4. Cells treated with 1 and 2 mM of polyP$_{700}$ exhibited only modest changes in metabolic activity over the experimental period, whereas 5 mM of polyP$_{700}$ slowed the increase in metabolic signal without causing a progressive decline consistent with excessive cytotoxicity. Additional assay controls, including wells containing cells without metabolic dye (blue) and media-only (pink) negative controls, confirmed expected assay performance throughout the 72-hour time course. Absorbance was measured at 460 nm. Data are representative of three independent experiments.
